# Supplementary material for: The automated optimisation of a coarse-grained force field using free energy data
Source: Phys Chem Chem Phys. 2021 Oct 19;23(43):24842–51. doi: 10.1039/d0cp05041e (PMC8579472; doi:10.1039/d0cp05041e)
Supplement: CP-023-D0CP05041E-s001 [file CP-023-D0CP05041E-s001.pdf]

Electronic Supplementary Material (ESI) for Physical Chemistry Chemical Physics.
This journal is © the Owner Societies 2021

Supporting Information for:

The Automated Optimisation of a Coarse-Grained Force Field Using Free Energy Data

Javier Caceres-Delpiano‡, Lee-Ping Wang†, Jonathan W. Essex‡*.*

‡ School of Chemistry, University of Southampton, Southampton, SO17 1BJ, United Kingdom.

† Department of Chemistry, University of California, Davis, California 95616, United States.

ForceBalance optimisation procedure. A trust region method approach has been used in the ForceBalance optimisations in this work. In trust region methods, there is a region of search space in which it is assumed the local derivative information is a good approximation of the objective function being minimised. After each optimisation step, the trust radius may be increased or decreased based on the quality Q of the steps taken, i.e. the ratio of the objective function change between steps i and i+1 and the expected change from the local derivative information at step i. The following formula is used to adjust the adaptive trust radius after the step is taken:

$$R_{i+1} = \max\left(R_{\min}, \frac{R_i}{1+a}\right) \qquad Q < 0.25\#(1)$$

$$R_{i+1} = R_i\left[1 + a\exp\left[-b\left(\frac{R_i}{R_0} - 1\right)\right]\right] \qquad Q > 0.75\#(2)$$

Here R_i is the current trust radius; R_{i+1} the trust radius at the next iteration; R_0 the default trust radius, was set to 0.1; and $R_{\min}$ the minimum trust radius, was set to 0.05. The parameter a, called "adapt_fac" in ForceBalance, which is related to how much the step size is increased, was set to 1.0; b, called "adapt_damp", that ties down the trust radius, was set to 0.2. The exponential term biases the current trust radius toward the default value, i.e. the trust radius increases by larger factors if the current value is smaller than the default, and vice versa if larger.

ForceBalance optimisation based on hydration free energy gradients. The optimisation of side-chain analogues and the protein backbone has been made based on atomistic hydration free energies, following 4 stages.

Stage A: Hydration free energy calculations on AT side-chain analogues, CG side-chains and backbone beads. The interaction energy terms between the solute and solvent are linearly related to the coupling parameter α. With this, the solvation free energies for the side-chain analogues, for the atomistic and coarse-grained systems, were calculated based on a decoupling approximation. That is, interactions between the solute and the solvent were gradually turned off. Our reference state will be our system in solution, and the final state will be the solute in vacuum. The OPLS-AA[1] and the AMBER-14SB[2] force fields were used for the atomistic side-chain analogues and the backbone, respectively. In all cases, systems were solvated in a TIP3P[3] water box. Since we are comparing our calculations with previous studies, especially the ones that give closer results to experiment, we have tried to be consistent with those, hence the choice of different force fields in the optimisation process. The SIRAH protein force field[4] was used for the CG side-chains and backbone beads, solvated in a WT4[5] water box. Electrostatic and van der Waals interactions were turned off together. Eleven discrete values of the coupling parameter α were used for the scaling of both CG and AT side-chain analogues potentials (see figure 2 and table S1 for details on the analogues used): 0.0, 0.1, 0.2, 0.3, 0.4, 0.5, 0.6, 0.7, 0.8, 0.9 and 1.0, where 0.0 and 1.0 represent the fully on and fully off systems. In the case of N-methylacetamide (NMA), which was used as a representation of the backbone beads, twenty-five values were used: 0.0, 0.5, 0.1, 0.15, 0.2, 0.25, 0.3, 0.35 0.4, 0.45, 0.5, 0.55, 0.6, 0.65, 0.7, 0.75, 0.8, 0.85, 0.87, 0.89, 0.91, 0.93, 0.95, 0.97 and 1.0. The soft-core scaling methods for Lennard-Jones (with $\alpha_{LJ} = 0.5$) and Coulombic interactions were used to smoothly vary the potentials[6,7]. Simulations were run for 5 ns per window, with a previous equilibration of 1 ns and 5000 iterations of the steepest descent algorithm. All the simulations were run using the NPT ensemble. The Multistate Bennett Acceptance Ratio (MBAR)[8] was used to compute the free energy difference, which combines data from multiple states. This

method is an extension of the well-known Bennett Acceptance Ratio (BAR)[9], which needs information from two states (in contrast to FEP[10] or TI[11], which need information from only one state) in order to compute the free energy difference.

For the AT simulations, a leap-frog stochastic dynamics integrator was used for integration of Newton's equations of motion with a time-step of 2 fs. Electrostatics interactions were calculated using the PME procedure[12] with a real-space cut-off of 1.2 nm and a Fourier grid spacing of 0.12 nm. Van der Waals interactions were modelled using the classical Lennard-Jones potential with a cut-off of 1.2 nm. The LINCS algorithm[13] was applied to constrain all H-bond lengths. AT simulations were run at 1 atm with the Parrinello-Rahman barostat[14] and at 298.15 K with the Berendsen thermostat[15].

For the CG simulations, a leap-frog stochastic dynamics integrator was used for integration of Newton's equations of motion with a time-step of 20 fs. Electrostatic interactions were calculated using the PME procedure with a grid spacing of 0.2 nm. Non-bonded interactions were modelled using the classical Lennard-Jones potential and a Coulombic energy function, with a cut-off of 1.2 nm each. All simulations were run at 1 atm with the Parrinello-Rahman barostat and at 298.15 K with the v-rescale thermostat[16]. All simulations were run with GROMACS v. 2018.2[17].

Stage B: Collection of AT $<\Delta U>_{\alpha}$ values. $<\Delta U>_{\alpha}$ were collected from the AT simulations in stage A, at different α values. For most of the side-chains, α simulations at 0.0 were not used due to the large magnitudes of $<\Delta U>_{\alpha}$ values and differences between AA and CG that could not be closely fitted. Val, Cys and Trp were the only exceptions for this case. $<\Delta U>_{\alpha}$ values were collected with an in-house Python code created for this purpose, averaging ΔU values for each

frame in the trajectories. Table S1 summarise the α values used for each of the simulated side-chains in the ForceBalance optimisation.

Stage C: Optimisation of SIRAH CG side-chains and backbone. Derivatives of the free energy gradients with respect to the parameters are calculated. These are used to build an objective function, which is a squared sum of the differences between the AA and CG $\langle \Delta U \rangle_\alpha$ values. The optimisation was carried out using ForceBalance using the same settings described in the WT4 model development, except for the adapt_fac and adapt_damp parameters, that were set to 0.2 and 0.5 respectively. Only 10 sets of parameters were optimised, 9 of them corresponding to 13 un-charged amino acid side-chains, as some of the side-chains are described by identical parameters, and 1 set corresponding to the backbone beads. In this case, the targets were atomistic free energy gradients at 2 or 3 different α simulation values (table S1). Proline is the only side-chain that has not been optimised given the lack of side-chain analogues, keeping its original parameter values. Only non-bonded parameters were optimised, including van der Waals epsilon (ε) values, and charges, mainly given the parameter sensitivity observed (see below for a discussion on parameter dependence and figure S1). All new optimised parameters are shown in table S2. All the optimisation simulations for the SIRAH beads were run with the optimised WT4-FB model (this work). 100 optimisation cycles were carried out, and the optimal parameters were taken from the lowest value of the objective function. Systems were minimised for 5000 steps using a steepest descent algorithm followed by an NPT equilibration time of 5 ns. Production runs were performed for 10 ns. A leap-frog algorithm was used for integration of Newton's equations of motion with a time-step of 20 fs. Electrostatic interactions are calculated using the Particle Mesh Ewald method[12] with a direct cut-off of 1.2 nm and a grid spacing of 0.2 nm. A 1.2 nm cut-off was used for van der

Waals interactions. The v-rescale thermostat[16] and the Parrinello-Rahman barostat[14] were used to maintain the temperature at 298.15 K and the pressure at 1 atm, respectively. The simulation conditions were consistent with the original SIRAH publication[4]. All simulations were run with GROMACS v. 2018.2[17]. All specific non-bonded pairs, previously set to the original SIRAH force field, between the backbone beads (GC, GN and GO) and water beads (WT) have been removed, and we have set those interactions using Lorentz-Berthelot combining rules.

Stage D: Re-calculation of CG hydration free energies. The optimised SIRAH-OBAFE force field was used for the re-calculation of the coarse-grained hydration free energies. The same protocol in stage A was used, with some minor differences based on the original publication of the SIRAH protein force field[4]. For all simulation, a leap-frog stochastic dynamics integrator was used for integration of Newton's equations of motion with a time-step of 20 fs. Electrostatic interactions were calculated using the PME procedure with a grid spacing of 0.2 nm. Non-bonded interactions were modelled using the classical Lennard-Jones potential and a Coulombic energy function, with a cut-off of 1.2 nm each. All simulations were run at 1 atm with the Parrinello-Rahman barostat[14] and at 298.15 K with the v-rescale thermostat[16]. All simulations were run with GROMACS v. 2018.2[17]. The new hydration free energies are shown in figure 2 and table S3, and they are compared with hydration free energies calculated from atomistic systems, with the SIRAH 1.0 and the updated SIRAH 2.0 protein force fields.

Hydration free energies of charged side-chains. Raw hydration free energies (equation 5) have been calculated using a lattice-summation scheme (PME) by decoupling the interactions, electrostatic and van der Waals together, of the ion (side-chain) with the solvent (excluding intramolecular interactions). Eleven lambda values have been used (0.0, 0.1, …, 0.9, 1.0) for all

the charged side-chains, using the GROMOS 54A8[18,19] for atomistic systems, the original SIRAH 1.0, the updated SIRAH 2.0, and SIRAH-OBAFE force fields in GROMACS v.2018.2. The simulation conditions and soft-core potential settings were similar to the ones used in the calculation of hydration free energies for uncharged side-chains (Stage A from the workflow in figure 1). A standard state correction was used with a value equal to 1.9 kcal•mol^{-1} for water, using a density value of 997 kg•m^{-3} (see refs 18, 20, 21). All the reported raw free energies exclude the self-interaction energy.

The corrections for the calculation of hydration free energies for charged are summarised below (see refs 18, 20, 21 for more details):

(A) Approximate representation of the electrostatic interactions (non-Coulombic) which lead to a deviation of the solvent polarization around the ion relative to an idealised Coulombic system, with also incomplete interactions of the ion with the solvent beyond the cut-off. This type A correction is specific for the electrostatic scheme used; it does not apply for lattice-summation schemes (PME), which are Coulombic in the limit of infinite system sizes, but it does apply for cut-off truncation (CT) or reaction field schemes (BM). The type A correction is specific for the electrostatic potential used, and is evaluated using the same potential, but in the idealized context of a macroscopic and non-periodic system. Moreover, it can be sub-divided into corrections A1 and A2 for CT schemes, which apply beyond the cut-off sphere of the ion and within it, respectively.

(B) Approximation of the size of the systems (finite), which do not follow a macroscopic regime. This leads to deviations on the solvent polarization, relative to the polarisation

of an ideal system (macroscopic). A clear example is the use of a computational box simulated under periodic boundary conditions. This type B correction is applied for the specific electrostatic scheme in the simulation (e.g., LS, CT or BM scheme).

(C) Deviation of the solvent generated electric potential at the atomic site of the ion relative to a "correct" electrostatic potential, which is a consequence of the use of an inappropriate summation scheme for the calculation of electrostatic interactions (i.e. P scheme, which stands for summing over individual charges, and a M scheme, which stands for summing over whole solvent molecules), as well as the presence of a constant potential offset. This type C correction is applied for a specific electrostatic scheme and choice of boundary conditions, and can be subdivided in type C_1 and C_2 corrections, for improper potential summation and for the potential offset, respectively.

(D) Approximate force-field representations, especially related to the wrong dielectric constant for the solvent model used.

Numerical solutions of the Poisson equation are needed to obtain an estimation of the charging free energy in an idealised system that obeys a macroscopic regime (non-periodic with Coulombic electrostatic interactions) and based on the experimental solvent permittivity (ΔG^{NPBC}_{chg}). Simulations of a periodic systems with a specific electrostatic scheme and based on the model solvent permittivity are also needed ($\Delta G^{PBC,LS}_{chg}$ for a periodic boundary condition system using a LS scheme). The sum of corrections A, B and D can be obtained based on these two continuum-electrostatic simulations, as

$$\Delta G^{LS}_{A+B+D} = \Delta G^{NPBC}_{chg} - \Delta G^{PBC,LS}_{chg} \#(S1)$$

for a LS scheme. The two terms on the right side of equation S1 are charging free energies obtained with the Poisson equation solver from references[22-24], for non-periodic and periodic systems with Coulombic electrostatic interactions, respectively.

In this work, a relative permittivity of 78.4 for water has been used in the calculation of ΔG_{chg}^{NPBC} A relative permittivity of 63.84 for the optimised WT4 water model was used, as calculated based in reference 26, in the calculations of $\Delta G_{chg}^{PBC,LS}$. Continuum-electrostatic calculation were done with the GROMOS++ pre-MD and analysis software v.1.4.1[25] and were based on single structure taken from as the final configuration of the hydration free energy simulations of the charged side-chains. The appropriate boundary conditions and electrostatic scheme were used for each case, with a grid spacing of 0.02 nm and a threshold of 10^{-6} kJ•mol^{-1} for the convergence of the electrostatic free energy.

Type C_1 correction is required for LS and BM (reaction field) schemes, and corrects the P-summation (atom-based cut-off) implied by these schemes to a proper M-summation (molecule-based cut-off). For a LS scheme, this is given by:

$$\Delta G_{C_1}^{LS} = - N_A q_i \left(1 - \frac{V_i}{\langle L \rangle^3} \right) \xi'_S \#(S2)$$

where N_A is the Avogadro's constant, V_i is the ionic volume (assumed constant and defined as the change in the volume of the computational box upon insertion of the neutral ion-sized cavity)[18], L is the length of the edge of the box, q_i is the total ionic charge, and ξ'_S corresponds to the exclusion

potential of the solvent model. For fully rigid models with a single van der Waals interaction site, this last term has been usually calculated based on the quadrupole moment trace of the solvent model. For more complex solvent models, different methods have been derived for the calculations of their exclusion potentials[26]. In this work, we have employed method IV from reference 26, which relies on the comparison of the raw potentials within a cavity using two different electrostatic schemes, assuming that the corrected potentials are equal. For this, we have used a cut-off truncation (CM) and reaction field schemes (BM). The difference in the raw potentials are related to ξ'_S as:

$$\xi'_S = -\left[\frac{2\left(\epsilon'_S - 1\right)}{2\epsilon'_S + 1}\left(1 - \frac{R_I^3}{R_C^3}\right)\right]^{-1}\left(\phi^{*,raw,CM} - \phi^{*,raw,BM}\right)\#\#(S3)$$

where R_I is the effective ionic radius, R_C is the cut-off, $\phi^{*,raw,CM}$ and $\phi^{*,raw,BM}$ are the raw electrostatic potentials within an uncharged cavity of the size of a CG sodium ion, and ϵ'_S corresponds to the dielectric permittivity of the solvent model, which has been calculated based on the methodology used in reference 26. Simulations of an un-charged sodium ion solvated in the optimised WT4-FB model were run for 1 ns using a BM scheme, with a reaction field permittivity ϵ_{RF} equal to 80. Electrostatic potentials at the cavity were obtained for both CM and BM schemes based on the electrostatic interaction of the cavity with the solvent within the cut-off R_C, using an *in-house* Python script created for this purpose. Simulation settings were similar to the previous one used in this work. The dielectric permittivity calculated here differs with the value previously reported in table 1, but is within the error. Moreover, it has been reported that dielectric permittivities calculated using a reaction-field scheme are more sensitive to the choice of simulation parameters such as the non-bonded cut-off. Given this, the lack of agreement is not

unexpected, but as a matter of consistency with previous studies[26], we decided to use the dielectric permittivity calculated in this section for the evaluation of the exclusion potential. Moreover, the dielectric permittivity for the WT4 water model calculated in this section is similar to the one calculated by Reif et al. [26] with a reported value of 66.7 using the SPC model.

Type C_2 corrections correct for the presence of an interfacial potential at the ion surface. This term is proportional to the ratio of the ionic volume to the box volume. With this, its magnitude is very small for the systems used in this work, and has been neglected in the calculation of the corrected hydration free energies.

Optimisation of charged side-chains. All the optimisation simulations for the SIRAH beads were run with the optimised WT4-FB model (this work). 100 optimisation cycles were run. Systems were minimised for 5000 steps using a steepest descent algorithm followed by an NPT equilibration time of 5 ns. Production runs were performed for 10 ns. A leap-frog algorithm was used for integration of Newton's equations of motion with a time-step of 20 fs. Electrostatic interactions are calculated using the Particle Mesh Ewald method[12] with a direct cut-off of 1.2 nm and a grid spacing of 0.2 nm. A 1.2 nm cutoff was used for van der Waals interactions. The v-rescale thermostat[16] and the Parrinello-Rahman barostat[14] were used to maintain the temperature at 298.15 K and the pressure at 1 atm, respectively. The simulation conditions were consistent with the original SIRAH publication[4]. All simulations were run with GROMACS v. 2018.2[17].

Table S1. α simulation values used for the collection of $<\Delta U>_\alpha$ values, that correspond to the targets in the optimisation of the CG beads in ForceBalance. Atomistic analogues used are shown in parenthesis.

**Side-chain**	**α values**
Asn (acetamide)	0.1, 0.2, 0.5
Cys (methanethiol)	0.0, 0.2, 0.4
His (methylimidazole)	0.1, 0.2, 0.4
Met (methyl-ethylsulfide)	0.1, 0.2, 0.4
Phe (toluene)	0.5, 0.6
Ser (methanol)	0.1, 0.2, 0.5
Trp (methylindole)	0.0, 0.4, 0.5
Tyr (*p*-cresol)	0.1, 0.4, 0.5
Val (propane)	0.0, 0.5
Backbone (N-methylacetamide)	0.1, 0.3

Parameter dependence. Initially, a screening test was performed to evaluate the parameter dependence of $<\Delta U>_\alpha$ with respect to the force field parameters, i.e. to evaluate the changes in $<\Delta U>_\alpha$ based on changes in the force field parameters. For some of the cases (Ser, Asn and Val), both van der Waals sigma (σ) and epsilon (ε) values were optimised in a first attempt. Based on the parameter dependence observed in figure S2 for the case of Val, the $<\Delta U>_\alpha$ values do not significantly change within a sensible range of van der Waals σ values. On the contrary, an important parameter dependence is shown with respect to the van der Waals ε values (i.e. big changes in $<\Delta U>_\alpha$ are observed when changes in the parameters are performed). In figure S2, the van der Waals parameters are plotted in the form of internal optimisation variables in ForceBalance

("mathematical parameters"), which are related to the physical parameters (i.e. the parameters that are actually printed in the force field file) as a shifted displacement form the original value:

$$K_{phys} = K_{phys0} + SF * K_{math} \#(S4)$$

where K_{phys} corresponds to the parameter that is used in the simulation after the optimisation process, K_{phys0} is the initial parameter before the optimisation, SF is the scaling factor and K_{math} the mathematical value used in the optimisation process. Finally, only van der Waals ε values and charges were optimised given the parameter sensitivity that exists (see table S2 and figure S2).

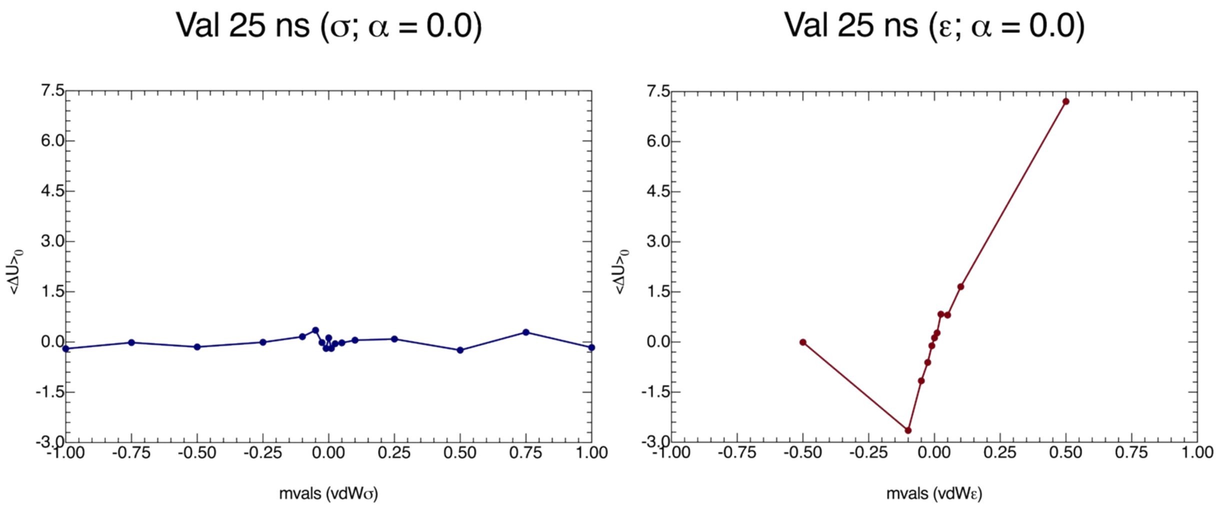

Figure S1. Parameter dependence for Val. Changes of $<\Delta U>_0$ (in units of $kcal \cdot mol^{-1}$) with respect to the van der Waals (vdW) σ and ε values are shown (left and right panel, respectively). Simulations were run at $\alpha = 0.0$ (fully on solute) for 25 ns. The simulation conditions were the same as the ones used for the side-chain optimisations (see stage A). Van der Waals values are plotted as mathematical values (mvals).

Atomistic gradient choice. It is important to note that for most cases, AT gradients at α=0 were too high to be fitted by ForceBalance due to the large magnitude of the gradient and the large difference between AT and CG. Inclusion of the α=0.0 point would have introduced a very large contribution to the objective function and worsened the quality of fit of all the other α points. We are assuming that the gradients should behave in a similar manner between the all-atom and coarse-grained systems, but this might not be the case. Using the free energy gradients as a proxy for the free energies, instead of the free energy itself, relies on the assumption that 1) if one of the free energy gradients is correct, we expect a better performance across the whole range of α values, and 2) coarse-grained and atomistic systems should have similar free energy gradients. Neither of these is necessarily true.

Special cases. The optimisation of methionine is an example where our method has worked (Fig. S2), finding a minimum, i.e. the optimal set of parameters to minimise the objective function. A manual search of 441 parameter combinations shown in figure S2 led us to similar results to those obtained for the full optimisation of methionine, with values of vdWσ = 0.49 nm, vdWε = 4.56 kJ·mol^{-1}, and vdWσ = 0.48 nm and vdWε = 4.22 kJ·mol^{-1}, respectively. Figure S3 shows the free energy gradients for the atomistic and coarse-grained methionine side-chain. The overall shape of the profile is maintained, but differences exists in the magnitude of the gradients. This may account for the differences observed for the calculated HFEs. Fortuitously, the optimised parameters led to better agreement with experimental hydration free energies.

In the case of phenylalanine, the optimised SIRAF-OBAFE parameters performed worse compared to the original SIRAH force fields, with values of 0.50 ± 0.05 kcal·mol^{-1} for the SIRAH 1.0 force field, 0.57 ± 0.05 kcal·mol^{-1} for the SIRAH 2.0 force field, vs. 1.12 ± 0.06 kcal·mol^{-1} for

the optimised SIRAH-OBAFE. We believe this is mainly due the complexity on the free energy gradient profile for this residue. Moreover, later optimisation runs of side-chains that share the A2C bead-types with phenylalanine (such as His, Tyr, and Trp) were performed using this parameter fixed to its original value.

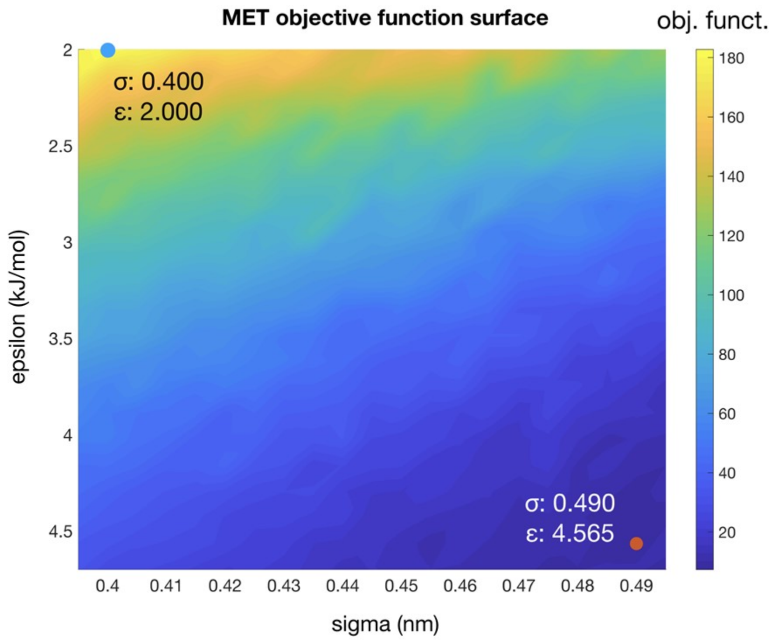

Figure S2. Methionine objective function surface. 441 combinations (21 x 21) of vdWσ and vdWε simulations were performed, and single calculations of the objective function were extracted and plotted. The maximum and minimum values for the objective function are shown as blue and orange dots, respectively, and for each of these the parameter combination is shown.

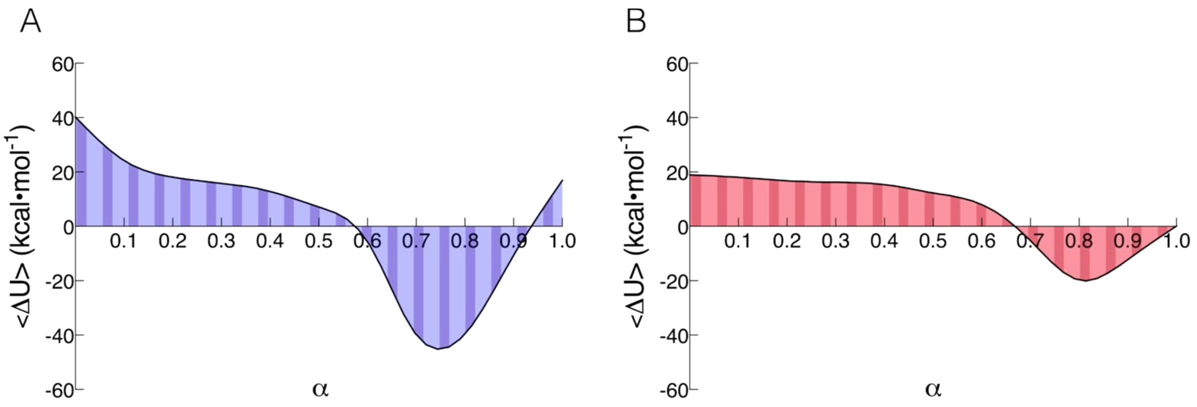

Figure S3. Free energy gradients for methionine. (A) atomistic free energy gradients and (B) coarse-grained free energy gradients. The results represent 11 α simulations with average $\langle \Delta U \rangle_\alpha$ values for each of those simulations shown.

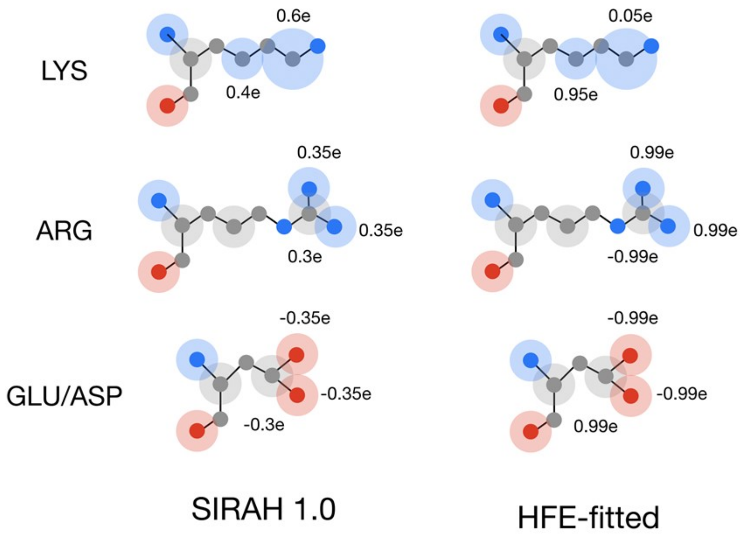

Figure S4. HFE-fitted charge values for the optimised charged side-chain. Schematic representation of the three optimised charged side-chains (Lys, Arg and Glu/Asp), for the original SIRAH 1.0 and the HFE-fitted parameter set (after the initial optimisation).

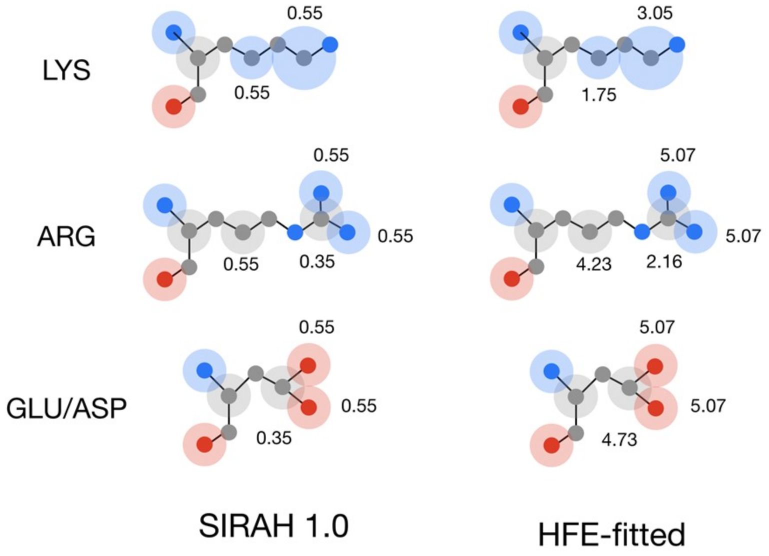

Figure S5. HFE-fitted VDWε values for the optimised charged side-chain. Schematic representation of the three optimised charged side-chains (Lys, Arg and Glu/Asp), for the original SIRAH 1.0 and the HFE- fitted parameter set (after the initial optimisation). All values are in units of kJ·mol^{-1}.

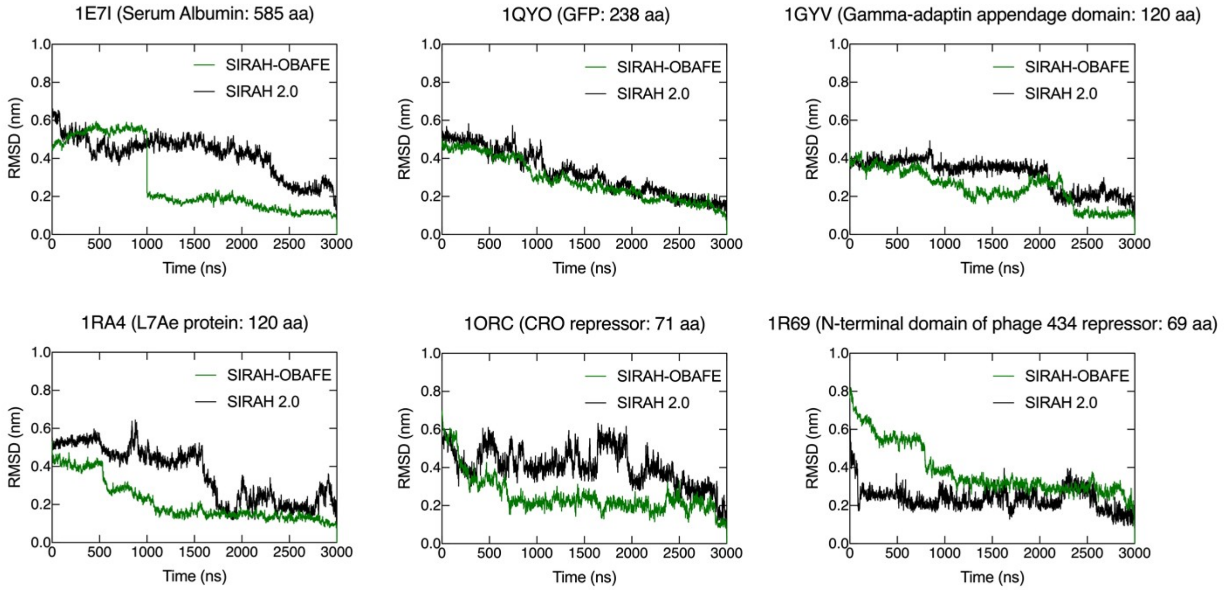

Figure S6. RMSD times series against the last frame. RMSD trajectory analysis is shown as a time series comparison with respect to the Cα carbons of the CG representation to the last frame of the trajectory for (A) Serum albumin, (B) GFP protein, (C) Gamma-adaptin domain, (D) L7Ae Archeal ribosomal protein, (E) CRO repressor and (F) the N-terminal domain of phage 434 repressor. PDB codes are shown in the figure titles. Simulations were run using the SIRAH 2.0 (black) and SIRAH-OBAFE (green) force fields.

Table S2. Optimised parameters for the SIRAH-OBAFE and WT4-FB force fields

	Bead type[a]	VDWσ (nm)	VDWε (kJ·mol^{-1})	Charge (*e*)
**Side-chain beads (SIRAH-OBAFE)**				
Asn/Gln	P3Cn/q	SaO[b]	3.5217E-01	0.00
	P5N	SaO	5.5453E-01	5.9527E-01
	P4O	SaO	5.547E-01	-5.9527E-01
Cys	P1S	SaO	1.0547E+00	-6.0817E-01
	P2P	SaO	2.2622E-01	6.0817E-01
His (epsilon protonated)	A2C	SaO	SaO	0.00
	A5E	SaO	1.7084E+00	5.0449E-01
	A5D	SaO	1.7023E+00	-5.0449E-01
Met	Y3Sm	SaO	4.7181E+00	0.00
Phe	A2C	SaO	SaO	0.00
	A1C	SaO	SaO	0.00
Ser/Thr	P1O	SaO	4.4658E-01	-9.1874E-01
	P2P	SaO	2.2622E-01	9.1874E-01
Trp	A2C	SaO	SaO	0.00
	A7N	SaO	6.9916E-01	-3.5323E-01
	A8P	SaO	1.5469E+00	3.5323E-01
	A1Cw	SaO	3.1449E+00	0.00
Tyr	A2C	SaO	SaO	0.00
	A4O	SaO	2.0418E+00	-3.5107E-01
	A3P	SaO	2.0491E+00	3.5107E-01
Val/Leu/Ile	Y4Cv/Y1C	SaO	5.0887E-01	0.00
Backbone	GC	SaO	5.5058E-01	4.2176E-01
	GO	SaO	5.2511E-01	-6.7336E-01
	GN	SaO	5.5058E-01	2.5161E-01

Arg	C2Cr	SaO	SaO	SaO
	C3Cr	SaO	SaO	SaO
	C5N	SaO	SaO	SaO
Lys	C1Ck	SaO	SaO	SaO
	C7Nk	SaO	SaO	SaO
Asp/Glu	C4Ce/d	SaO	SaO	SaO
	C6O	SaO	SaO	SaO
**WT4-FB**				
	WN1	4.2474E-01	7.6717E-01	-2.6730E-01
	WN2	4.2474E-01	7.6717E-01	-5.6223E-01
	WP1	4.2474E-01	7.6717E-01	2.6730E-01
	WP2	4.2474E-01	7.6717E-01	5.6223E-01

[a] Bead types taken from the original SIRAH publication[4].

[b] SaO, same as original, taken from the SIRAH protein force field publication[4].

Table S3 Hydration free energies of neutral side-chains and backbone using the OPLS-AA, AMBER-14SB, SIRAH 1.0, SIRAH 2.0 and SIRAH-OBAFE force fields[a,b]

	**Expt.**	**OPLS-AA[c]**	**SIRAH 1.0[c]**	**SIRAH 2.0[c]**	**SIRAH-OBAFE[c]**
Backbone (NMA)	10.1	7.40 ± 0.04 (AMBER-14SB)	-1.73 ± 0.07	-0.16 ± 0.05	10.91 ± 0.05
Val (propane)	-1.99	-2.45 ± 0.06	-0.02 ± 0.01	-0.18 ± 0.01	-2.26 ± 0.03
Leu (isobutane)	-2.28	-2.69 ± 0.10	-0.02 ± 0.01	-0.18 ± 0.01	-2.26 ± 0.03
Ile (butane)	-2.15	-2.59 ± 0.08	-0.02 ± 0.01	-0.18 ± 0.01	-2.26 ± 0.03
Ser (methanol)	5.06	4.44 ± 0.01	-1.87 ± 0.04	0.10 ± 0.09	5.26 ± 0.10
Thr (ethanol)	4.88	4.12 ± 0.11	-1.87 ± 0.04	-0.40 ± 0.01	5.26 ± 0.10
Cys (methanethiol)	1.24	0.39 ± 0.02	-1.78 ± 0.03	-0.71 ± 0.01	0.92 ± 0.07
Met (methyl-	1.48	0.06 ± 0.01	-0.03 ± 0.02	-0.01 ± 0.03	1.36 ± 0.02

ethylsulfide)					
Asn (acetamide)	9.68	8.46 ± 0.02	2.87 ± 0.07	2.85 ± 0.04	8.12 ± 0.05
Gln (propionamide)	9.38	8.36 ± 0.04	2.87 ± 0.07	2.86 ± 0.04	8.12 ± 0.05
Phe (toluene)	0.76	0.40 ± 0.04	0.50 ± 0.05	0.57 ± 0.05	1.12 ± 0.06
Tyr (*p*-cresol)	6.11	4.61 ± 0.13	0.70 ± 0.06	0.67 ± 0.02	5.10 ± 0.04
His (methyimidazole)	10.27	7.70 ± 0.06	1.47 ± 0.04	1.24 ± 0.02	8.46 ± 0.08
Trp (methylindole)	5.88	5.55 ± 0.22	-0.47 ± 0.09	1.52 ± 0.02	4.51 ± 0.04
**MUE[b]**		**1.04**	**5.03**	**4.45**	**0.68**
**MSE[b]**		**-1.04**	**-4.13**	**-3.61**	**-0.43**
**R^2**		**0.98**	**0.10**	**0.40**	**0.97**

[a] Values are in the units of kcal·mol^{-1}. Experimental values were obtained from reference 27. OPLS-AA and AMBER-14SB values were re-calculated using the corresponding side-chain analogues listed in parenthesis, based on reference [26].

[b] Mean signed error (MSE), mean unsigned error (MUE) and determination coefficient (R^2).

[c] Error bars modelled as standard errors across three repeat simulations.

REFERENCES

(1) Jorgensen, W. L.; Tirado-Rives, J. The OPLS [Optimized Potentials for Liquid Simulations] Potential Functions for Proteins, Energy Minimizations for Crystals of Cyclic Peptides and Crambin. *J. Am. Chem. Soc.* **1988**, 110 (6), 1657–1666.

(2) Maier, J. A.; Martinez, C.; Kasavajhala, K.; Wickstrom, L.; Hauser, K. E.; Simmerling, C. ff14SB: Improving the Accuracy of Protein Side Chain and Backbone Parameters from ff99SB. *J. Chem. Theory Comput.* **2015**, 11 (8), 3696–3713.

(3) Jorgensen, W. L.; Chandrasekhar, J.; Madura, J. D.; Impey, R. W.; Klein, M. L. Comparison of Simple Potential Functions for Simulating Liquid Water. *J. Chem. Phys.* **1983**, 79 (2), 926–935.

(4) Darré, L.; Machado, M. R.; Brandner, A. F.; González, H. C.; Ferreira, S.; Pantano, S. SIRAH: a Structurally Unbiased Coarse-Grained Force Field for Proteins with Aqueous Solvation and Long-Range Electrostatics. *J. Chem. Theory Comput.* **2015**, 11 (2), 723–739.

(5) Darré, L.; Machado, M. R.; Dans, P. D.; Herrera, F. E.; Pantano, S. Another Coarse Grain Model for Aqueous Solvation: WAT FOUR? *J. Chem. Theory Comput.* **2010**, 6 (12), 3793–3807.

(6) Anwar, J.; Heyes, D. M. Robust and Accurate Method for Free-Energy Calculation of Charged Molecular Systems. *J. Chem. Phys.* **2005**, 122 (22), 224117.

(7) Shi, Y.; Wu, C.; Ponder, J. W.; Ren, P. Multipole Electrostatics in Hydration Free Energy Calculations. *J. Comput. Chem.* **2011**, 32 (5), 967–977.

(8) Shirts, M. R.; Chodera, J. D. Statistically Optimal Analysis of Samples From Multiple Equilibrium States. *J. Chem. Phys.* **2008**, 129 (12), 124105.

(9) Bennett, C. H. Efficient Estimation of Free Energy Differences From Monte Carlo Data. *J. Comput. Phys.* **1976**, 22 (2), 245–268.

(10) Zwanzig, R. W. High-Temperature Equation of State by a Perturbation Method. I. Nonpolar Gases. *J. Chem. Phys.* **1954**, 22 (8), 1420–1426.

(11) Kirkwood, J. G. Statistical Mechanics of Fluid Mixtures. *J. Chem. Phys.* **1935**, 3 (5), 300–313.

(12) Darden, T.; York, D.; Pedersen, L. Particle Mesh Ewald: an N·Log(N) Method for Ewald Sums in Large Systems. *J. Chem. Phys.* **1993**, 98 (12), 10089–10092.

(13) Hess, B.; Bekker, H.; Berendsen, H. J. C.; Fraaije, J. G. E. M. LINCS: a Linear Constraint Solver for Molecular Simulations. *J. Comput. Chem.* **1997**, 18 (12), 1463–1472.

(14) Parrinello, M.; Rahman, A. Crystal Structure and Pair Potentials: a Molecular-Dynamics Study. *Phys. Rev. Lett.* **1980**, 45 (14), 1196–1199.

(15) Berendsen, H. J. C.; Postma, J. P. M.; van Gunsteren, W. F.; DiNola, A.; Haak, J. R. Molecular Dynamics with Coupling to an External Bath. *J. Chem. Phys.* **1984**, 81 (8), 3684–3690.

(16) Bussi, G.; Donadio, D.; Parrinello, M. Canonical Sampling Through Velocity Rescaling. *J. Chem. Phys.* **2007**, 126 (1), 014101.

(17) Abraham, M. J.; Murtola, T.; Schulz, R.; Páll, S.; Smith, J. C.; Hess, B.; Lindahl, E. GROMACS: High Performance Molecular Simulations Through Multi-Level Parallelism From Laptops to Supercomputers. *SoftwareX* **2015**, 1-2, 19–25.

(18) Reif, M. M.; Hünenberger, P. H.; Oostenbrink, C. New Interaction Parameters for Charged Amino Acid Side Chains in the GROMOS Force Field. *J. Chem. Theory Comput.* **2012**, 8 (10), 3705–3723.

(19) Reif, M. M.; Winger, M.; Oostenbrink, C. Testing of the GROMOS Force-Field Parameter Set 54A8: Structural Properties of Electrolyte Solutions, Lipid Bilayers, and Proteins. *J. Chem. Theory Comput.* **2013**, 9 (2), 1247–1264.

(20) Reif, M. M.; Hünenberger, P. H. Computation of Methodology-Independent Single-Ion Solvation Properties From Molecular Simulations. IV. Optimized Lennard-Jones Interaction Parameter Sets for the Alkali and Halide Ions in Water. *J. Chem. Phys.* **2011**, 134 (14), 144104.

(21) Reif, M. M.; Hünenberger, P. H. Computation of Methodology-Independent Single-Ion Solvation Properties From Molecular Simulations. III. Correction Terms for the Solvation Free Energies, Enthalpies, Entropies, Heat Capacities, Volumes, Compressibilities, and Expansivities of Solvated Ions. *J. Chem. Phys.* **2011**, 134 (14), 144103.

(22) Davis, M. E.; Madura, J. D.; Luty, B. A.; McCammon, J. A. Electrostatics and Diffusion of Molecules in Solution: Simulations with the University of Houston Brownian Dynamics Program. *Comput. Phys. Commun.* **1991**, 62 (2-3), 187–197.

(23) Madura, J. D.; Briggs, J. M.; Wade, R. C.; Davis, M. E.; Luty, B. A.; Ilin, A.; Antosiewicz, J.; Gilson, M. K.; Bagheri, B.; Scott, L. R.; McCammon, J. A. Electrostatics and Diffusion of Molecules in Solution: Simulations with the University of Houston Brownian Dynamics Program. *Comput. Phys. Commun.* **1995**, 91 (1-3), 57–95.

(24) Hünenberger, P. H.; McCammon, J. A. Effect of Artificial Periodicity in Simulations of Biomolecules Under Ewald Boundary Conditions: a Continuum Electrostatics Study. *Biophys. Chem.* **1999**, 78 (1-2), 69–88.

(25) Eichenberger, A. P.; Allison, J. R.; Dolenc, J.; Geerke, D. P.; Horta, B. A. C.; Meier, K.; Oostenbrink, C.; Schmid, N.; Steiner, D.; Wang, D.; van Gunsteren, W. F. GROMOS++ Software for the Analysis of Biomolecular Simulation Trajectories. *J. Chem. Theory Comput.* **2011**, 7 (10), 3379–3390.

(26) Reif, M. M.; Hünenberger, P. H. Origin of Asymmetric Solvation Effects for Ions in Water and Organic Solvents Investigated Using Molecular Dynamics Simulations: the Swain Acity-Basity Scale Revisited. *J. Phys. Chem. B* **2016**, 120 (33), 8485–8517.

(27) Chang, J.; Lenhoff, A. M.; Sandler, S. I. Solvation Free Energy of Amino Acids and Side-Chain Analogues. *J. Phys. Chem. B* **2007**, *111* (8), 2098–2106.
